# Systematic analysis of the lysine malonylome in *Sanghuangporus sanghuang*

**DOI:** 10.1186/s12864-021-08120-0

**Published:** 2021-11-19

**Authors:** Tong Wang, Guangyuan Wang, Guoli Zhang, Ranran Hou, Liwei Zhou, Xuemei Tian

**Affiliations:** 1 Shandong Province Key Laboratory of Applied Mycology, Qingdao Agricultural University, Changcheng Road, No.700, Qingdao, 266109 China; 2 State Key Laboratory of Mycology, Institute of Microbiology, Chinese Academy of Sciences, Beijing, 100101 China

**Keywords:** Malonylation, Posttranslational modification (PTM), Malonylproteome, *Sanghuangporus sanghuang*

## Abstract

**Background:**

*Sanghuangporus sanghuang* is a well-known traditional medicinal mushroom associated with mulberry. Despite the properties of this mushroom being known for many years, the regulatory mechanisms of bioactive compound biosynthesis in this medicinal mushroom are still unclear. Lysine malonylation is a posttranslational modification that has many critical functions in various aspects of cell metabolism. However, at present we do not know its role in *S. sanghuang*. In this study, a global investigation of the lysine malonylome in *S. sanghuang* was therefore carried out.

**Results:**

In total, 714 malonyl modification sites were matched to 255 different proteins. The analysis indicated that malonyl modifications were involved in a wide range of cellular functions and displayed a distinct subcellular localization. Bioinformatics analysis indicated that malonylated proteins were engaged in different metabolic pathways, including glyoxylate and dicarboxylate metabolism, glycolysis/gluconeogenesis, and the tricarboxylic acid (TCA) cycle. Notably, a total of 26 enzymes related to triterpene and polysaccharide biosynthesis were found to be malonylated, indicating an indispensable role of lysine malonylation in bioactive compound biosynthesis in *S. sanghuang*.

**Conclusions:**

These findings suggest that malonylation is associated with many metabolic pathways, particularly the metabolism of the bioactive compounds triterpene and polysaccharide. This paper represents the first comprehensive survey of malonylation in *S. sanghuang* and provides important data for further study on the physiological function of lysine malonylation in *S. sanghuang* and other medicinal mushrooms.

**Supplementary Information:**

The online version contains supplementary material available at 10.1186/s12864-021-08120-0.

## Background

*Sanghuangporus sanghuang* (Hymenochaetaceae, Basidiomycota), an herbal mushroom, has been used for more than 2000 years in China. It was previously mistaken for *Inonotus linteus* or *Inonotus baumii* for a long time. In 2012, it was identified as a new species *Inonotus sanghuang* and in 2016, it was renamed *S. sanghuang* [1, 2]*.*

Extensive work has shown that *S. sanghuang* has a diverse range of biological activities [3–6]. The active compounds that play a major role in this medicinal fungus are triterpenoids and polysaccharides. However, the mechanism underlying the regulation of bioactive compound biosynthesis in *S. sanghuang* is still unclear.

Posttranslational modifications (PTMs) play a pivotal role in modulating different cellular pathways and disease processes, and over 400 distinct forms of PTMs have been found [7, 8]. Lysine malonylation is an evolutionarily conserved PTM. Malonylation has been reported to use malonyl-CoA as a substrate in protein modification [9]. However, we still know little about the enzymes that regulate the malonylation state of proteins [10]. To date, with advances in high-throughput experimental techniques, thousands of malonylated proteins have been discovered. These malonylated proteins have been found to be located in chloroplasts, the mitochondria, the cytoplasm, and the nucleus [10–15], suggesting that lysine malonylation is regulated in diverse metabolic processes.

Although malonoyl modifications have been studied in many species, few studies have focused on the mushroom malonylome. Similar to the effects in other organisms, such as mammals, plants, and bacteria [15, 16], we speculated that lysine malonylation may affect various metabolic processes in *S. sanghuang*. To demonstrate this hypothesis, we conducted a proteomics study of malonylated proteins in *S. sanghuang*. The results of this study provide a comprehensive view of the regulation of lysine malonylation in a wide range of biological processes, particularly in the biosynthesis of bioactive metabolites and secondary metabolites.

## Methods

### Fungal strain

The *S. sanghuang* CGMCC NO.21068 mycelia used in this study were isolated from fruit bodies collected from the mountainous area of Anshun city, Guizhou Province, China. The specimen was deposited in the Mycological Herbarium, Qingdao Agricultural University (HMQAU), Qingdao. In this study, the strain was stored at 4 °C in solid medium slants composed of 20 g/l bran, 30 g/l corn, 30 g/l glucose (catalogue #A501991, Sangon Biotech, China), 1 g/l KH_2_PO_4_ (catalogue #A100781, Sangon Biotech, China), 0.5 g/l MgSO_4_.7H_2_O (catalogue #A500864, Sangon Biotech, China), 4 g/l yeast extract (catalogue #A100850, Sangon Biotech, China), 3 g/l peptone (catalogue #A505247, Sangon Biotech, China) and 20 g/l agar (catalogue #A100637, Sangon Biotech, China). The strain was incubated on liquid medium (the medium was prepared as the solid medium mentioned-above but without the agar) at 26 °C and 150 rpm for 7 d. Then, the fermentation broth was filtered to collect the mycelia, flash frozen in liquid nitrogen, and stored at − 80 °C to be used for lysine malonylation analysis.

Morphological and molecular identification of the *S. sanghuang* strain was performed according to a previous study. The microscopic characteristics were studied under a Zeiss/Axioscope A1 microscope at magnifications of up to 1000×. The macroscopic and microscopic morphological characteristics were consistent with previous studies [17–19]. Phylogenetic analysis based on ITS sequence also confirmed that the strain was *S. sanghuang* [19, 20].

### Protein extraction and trypsin digestion

Protein extraction was performed as previously described [7, 10]. Briefly, the tissue samples were ground to powder in a precooled mortar with liquid nitrogen. A fourfold volume of extraction buffer containing 10 mM dithiothreitol (catalogue #D9163-5G, Sigma-Aldrich, USA), 1% protease inhibitor (catalogue #524633-1ML, Calbiochem, Merck, USA), 3 μM trichostatin A (catalogue #58880–19-6, Sigma-Aldrich, USA), and 50 mM nicotinamide (catalogue #N3376-100 g, Sigma-Aldrich, USA) was added and the cells were lysed by sonication using a ultrasonic processor (catalogue #JY92-N, Scientz, Ningbo, China) as previously described [10]. Equivalent volumes of Tris-equilibrated phenol were added. After centrifugation at 5500×g and 4 °C for 10 min (catalogue #5424R, Eppendorf, Germany), the supernatants were collected and sedimented overnight with a 5-fold volume of 0.1 M ammonium acetate (catalogue #73594, Sigma-Aldrich, USA), and the protein precipitate was washed with methanol (catalogue #34860, Sigma-Aldrich, USA) and acetone (catalogue #270725, Sigma-Aldrich, USA). Then, 8 M urea (catalogue #V900119-500G, Sigma-Aldrich, USA) was redissolved for precipitation. The protein concentration was measured using bicinchoninic acid (BCA) kits (catalogue #P0011–1, Beyotime Biotechnology, China). Finally, the extracted proteins were digested by trypsin (catalogue #V5111, Promega, Madison, USA) as according to previously described procedures [7, 10].

### HPLC fractionation and affinity enrichment

High-pH reverse HPLC fractionation was used for peptides on an Agilent 300 Extend C18 column (5 μm, 4.6 mm, 250 mm) (Agilent, Santa Clara, USA). The operation was performed as follows: sterilized peptide fractions were isolated in a gradient between 8 and 32% acetonitrile (catalogue #A998–4, Fisher Chemical, USA) (pH 9) for 60 min. They were merged into 4 fractions and freeze-dried under vacuum. The polypeptides were dissolved in IP buffer (100 mM NaCl (catalogue #S3014, Sigma-Aldrich, USA), 1 mM EDTA (catalogue #V900081-500 g, Sigma-Aldrich, USA), 50 mM Tris-HCl (catalogue #V900483-500G, Sigma-Aldrich, USA), and 0.5% NP-40 (catalogue #18896, Sigma-Aldrich, USA), pH 8.0) [10]. The polypeptides were incubated with pan anti-malonyllysine antibody conjugated agarose beads (catalogue #PTM-904, PTM Biotech, China) at 4 °C overnight. Finally, the bound peptides on the agarose beads were eluted three times with 0.1% trifluoroacetic acid (catalogue #58880–19-6, Sigma-Aldrich, USA), followed by desalting using C18 ZipTips (catalogue #Z720046, Merck Millipore, USA) [21–23].

### LC-MS/MS analysis

The obtained peptides were dissolved in 0.1% formic acid (catalogue #56302-50ML-F, Sigma-Aldrich, USA) and separated by ultrahigh-performance liquid chromatography (UHPLC) using an EASY-nLC 1000 (Thermo Scientific, USA). Mobile phase A consisted of 0.1% formic acid (catalogue #56302-50ML-F, Sigma-Aldrich, USA) and 2% acetonitrile (catalogue #A998–4, Fisher Chemical, USA). Mobile phase B consisted of 0.1% formic acid and 90% acetonitrile. The liquid phase gradient was set as follows: 0–20 min, 7–25% B; 20–34 min, 25–38% B; 34–37 min, 38–80% B; and 37–40 min, 80% B, with a flow rate of 500 nL/min.

After HPLC separation, the peptides were injected into a nanospray ionization (NSI) ion source for ionization and mass spectrometry (MS) analysis by a Q Exactive Plus instrument (Thermo Fisher Scientific, USA) [10]. The ion source voltage was set to 2.2 kV. The primary MS scanning range was 350–1800 m/z, and the secondary MS scanning range was 100.0 m/z. Data collection was performed using the data-dependent acquisition (DDA) procedure. The automatic gain control (AGC) was set to 5e4 [24], The dynamic rejection time was set to 15 s to avoid repeated scanning, the parameter threshold was set to 5e3 ions/s, and the maximum injection time was set to 200 ms.

### Database search

The obtained MS/MS data were analysed with MaxQuant software [25]. The *S. sanghuang* database was used (transcriptome, 23,290 sequences). Reverse libraries were added to calculate the false discovery rate (FDR), and contamination libraries were added to eliminate the effects of contaminating proteins. Trypsin/P was applied as the cleavage enzyme and the number of missed cleavages was set to 4 [10]. The first search and main search primary parent ion mass error tolerance was set to 20 ppm and 5 ppm, respectively. Cysteine alkylation was set as the fixed modification, and the variable modifications were acetylation of the protein N-terminus, deamidation of aspartyl/glutamyl groups, and malonylation of lysine. All the FDRs were set to 1% [26].

### Bioinformatics analyses

The Gene Ontology (GO) annotations of the proteins were classified into the biological process, cellular component, and molecular function categories [27]. The GO annotations of the malonylated proteins were from the UniProt-Gene Ontology Annotation (GOA) database (http://www.ebi.ac.uk/GOA/) [28]. InterProScan was used to annotate the domain functional domain descriptions of the malonylated proteins [29, 30]. The metabolic pathways associated with the modified proteins were analysed using the Kyoto Encyclopedia of Genes and Genomes (KEGG) database (https://www.genome.jp/kegg/). The subcellular localizations of the identified proteins were annotated using WoLF PSORT [31]. The significance of malonylated protein enrichment was measured by Fisher’s exact (two-sided) test and *p* value < 0.05 was considered to be significant [32–36]. To investigate the protein-protein interaction (PPI) network, all the modified proteins were searched against the STRING database [37]. Then, the visualization of the PPI network from STRING was presented with the R package “networkD3” [38, 39].

## Results

### Proteome-scale analysis of malonylated proteins in *S. sanghuang*

**Fig. 1:**
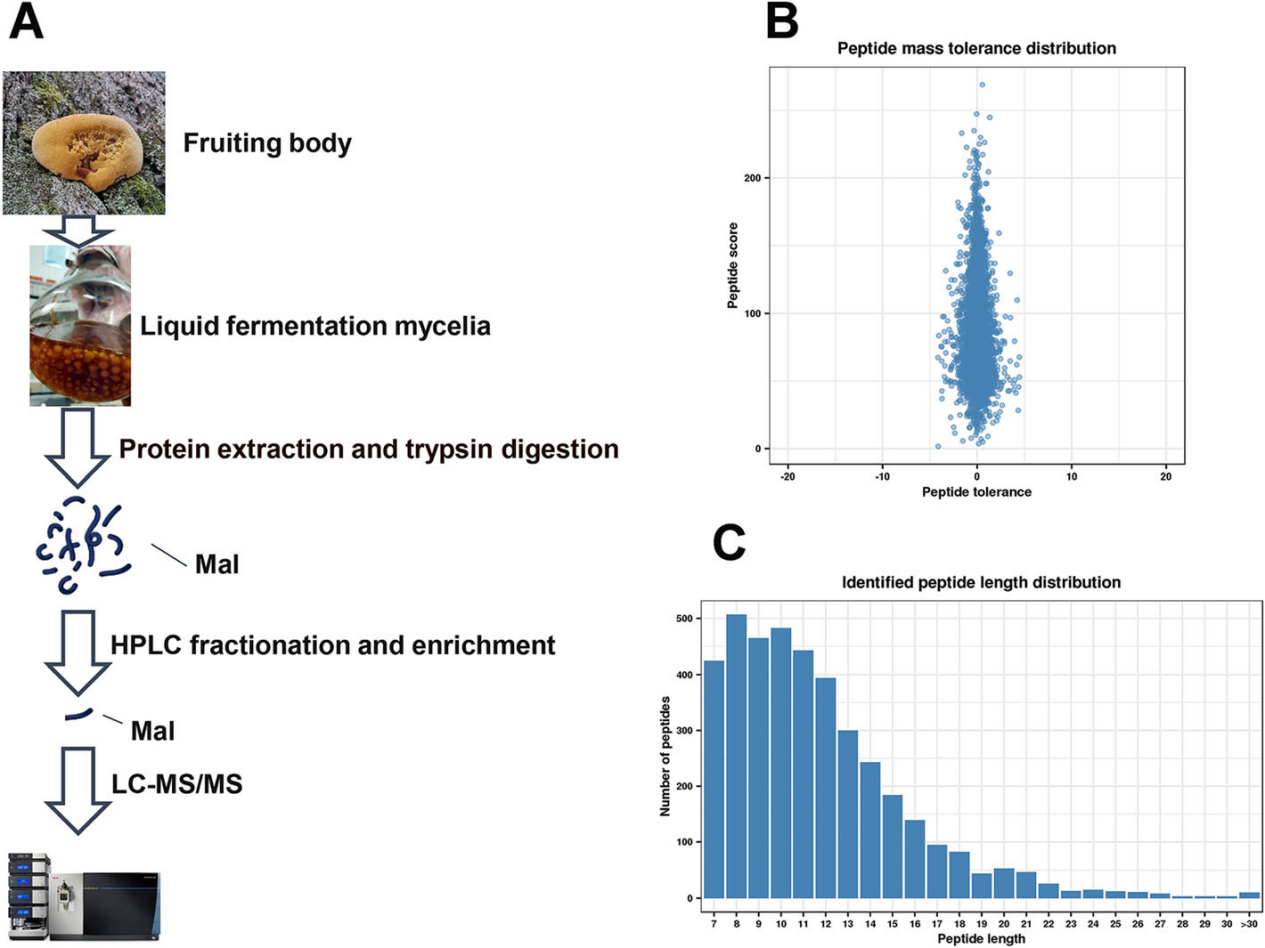
Analysis of malonylated sites in *S*. *sanghuang*. **a** Technology roadmap in this study. **b** Mass distribution of error for all malonyl peptides **c** Length distribution of modified peptides

In this project, a range of technologies, such as HPLC, malonylation peptide enrichment and MS-based proteomics technologies, were combined for qualitative proteomics of malonylation in *S. sanghuang* (Fig. 1a). The results showed that the peptide score was between − 10 and 10 (Fig. 1b). The tolerance of the peptides was in a reasonable range. The distribution of the identified peptide lengths was examined, and the lengths of most peptides were between 7 and 22 (Fig. 1c), meeting the requirements of proteomic analysis. The MS results of malonylated peptides are summarized in Additional file 1: Fig. S1. Finally, 713 malonyl-modified sites matched to 255 different proteins were identified in *S. sanghuang* (Additional file 2: Table S1). Among them, many were related to triterpene synthesis. Farnesyl pyrophosphate synthases (FPPs), which are are pivotal enzymes in the main pathway of triterpene synthesis, were found to be malonylated, indicating that lysine malonylation was involved in bioactive compound biosynthesis.

### Pattern analysis of malonylated sites

**Fig. 2:**
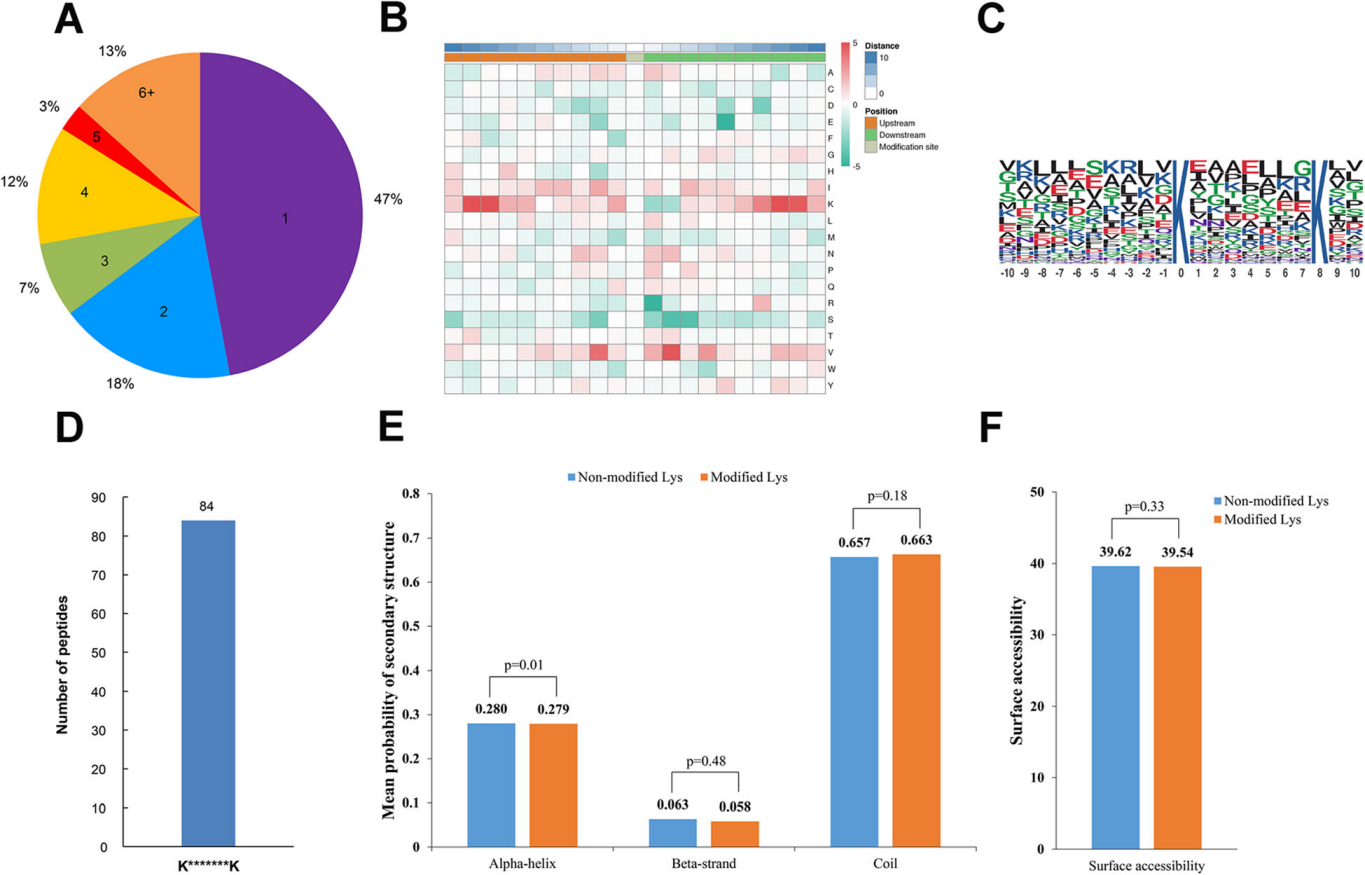
Characterization of malonylation sites. **a** Pie chart of the percentage and number of malonylated residues in the protein. **b** The frequency heat map of amino acid composition around malonylation. **c** Conservatism of malonylation sites. **d** Number of malonylation motif peptides. K indicates modified lysine sites and * denotes random amino acid sites. **e** Predictive analysis of the secondary structure of malonyl proteins. The Y-axis indicates the average probability of secondary structure, and the larger the value of Y, the higher the probability of this secondary structure configuration. **f** Peptides surface accessibility of malonylation sites

To evaluate the distribution of malonylation sites in *S. sanghuang*, the number of identified modification sites was calculated for each protein. As shown in Fig. 2a, 47% of the proteins had one malonylation site, while only 18, 7, 12, 3, and 13% of the proteins contained 2, 3, 4, 5, and 6 or more modification sites, respectively. It has been documented that modification is prioritized at specific lysine sites (Additional file 2: Table S3) [10]. Therefore, the compositional frequencies of the amino acids surrounding malonyl lysine were examined. As shown in Fig. 2b, lysine (K) had the highest frequencies in the − 10 to + 10 position, whereas arginine (R) and glutamate (E) had the lowest frequencies. Hence, proteins with this group are the preferred substrates for malonyltransferases in *S. sanghuang.* Consistent with the results of the motif enrichment heatmap (Fig. 2b), only one motif was detected (Fig. 2c,d). To elucidate the secondary structure of proteins and the correlation between modified lysines, the secondary structures of all the malonylated proteins in *S. sanghuang* were examined (Fig. 2e). More malonylation sites were located more in the coiled-coil regions (*p* = 0.18) than in the α-helical (*p* = 0.01) and β-strand (*p* = 0.48) regions, suggesting that malonylation may favour the disordered structures of *S. sanghuang.* In addition, we assessed the surface accessibility of malonylated lysine sites and found that 39.62% of the unmodified lysine residues were located on the protein surface, compared to 39.54% of the modified lysine sites (Fig. 2f). As such, the protein’s surface accessibility may be influenced by lysine malonylation.

### Functional annotation and cellular localization of malonylated proteins in *S. sanghuang*

**Fig. 3:**
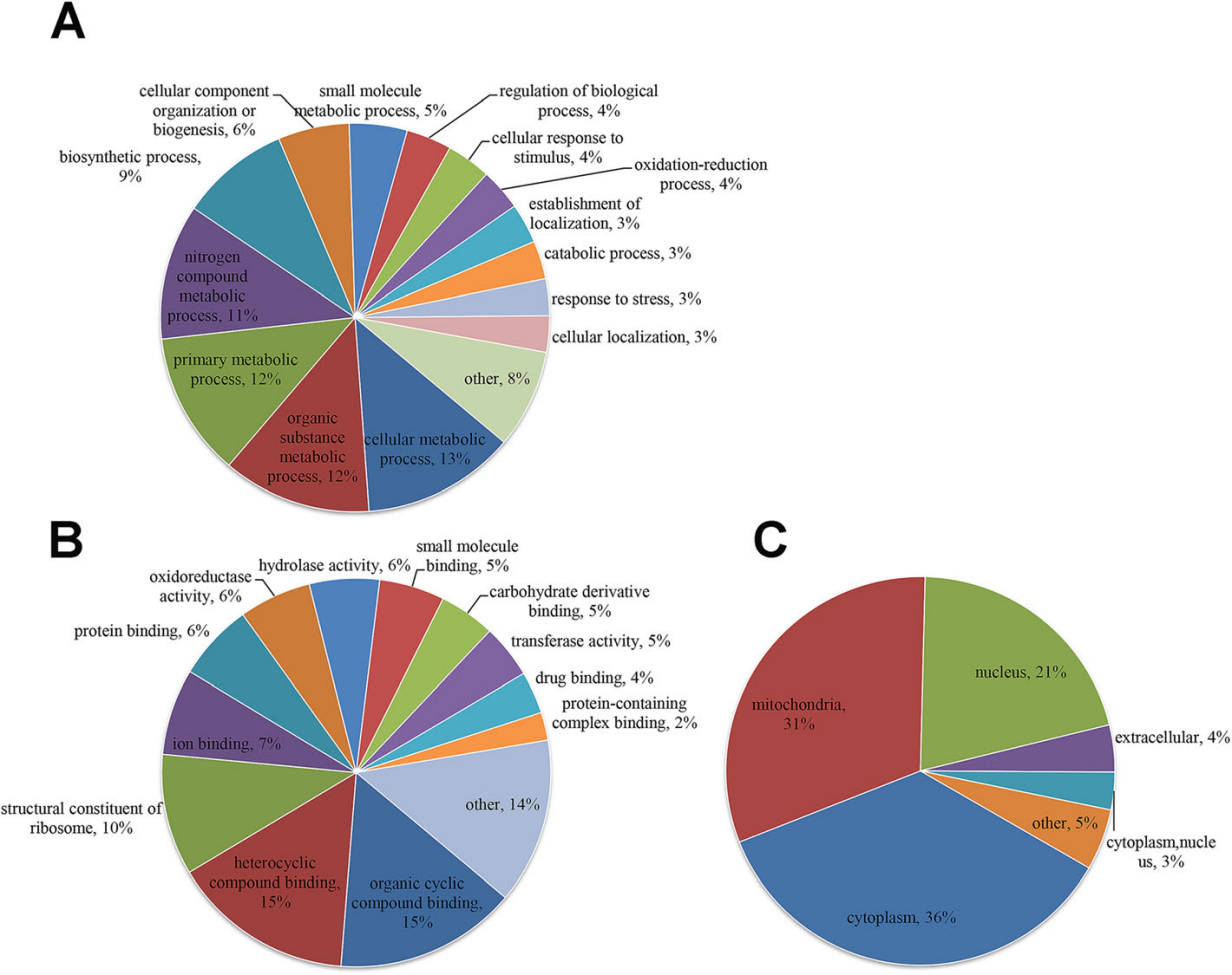
Functional classification of malonylated proteins in *S*. *sanghuang.* Each pie chart shows the percentage of malonylated proteins in each category. The GO annotation classifies proteins according to their biological processes and molecular functions. **a** Classification of malonylated proteins based on biological process **b** Classification of malonylated proteins based on molecular function. **c** Subcellular localization of the malonylated proteins

For better comprehension of the malonylated proteins in *S. sanghuang* and their corresponding biological processes and molecular functions, we annotated and classified the identified proteins. GO analysis showed that the malonylated proteins had extensive activity in molecular functions and biological processes in *S. sanghuang*. The most abundant group of malonylated proteins in the biological process category consisted of enzymes related to metabolism (53%) (Fig. 3a). The majority of the malonylated proteins were associated with organocyclic compound binding (15%), heterocyclic compound binding (15%) and structural constituent of ribosome (10%) within the molecular functional classification (Fig. 3b). Characterization of the subcellular localization of the malonylated proteins showed that the modified proteins were found in the cytoplasm (36%), mitochondria (31%), and nucleus (21%) (Fig. 3c). These observations show that malonylated proteins have multiple functions and are widely present in *S. sanghuang.*

#### Functional enrichment analysis of malonylated proteins

**Fig. 4:**
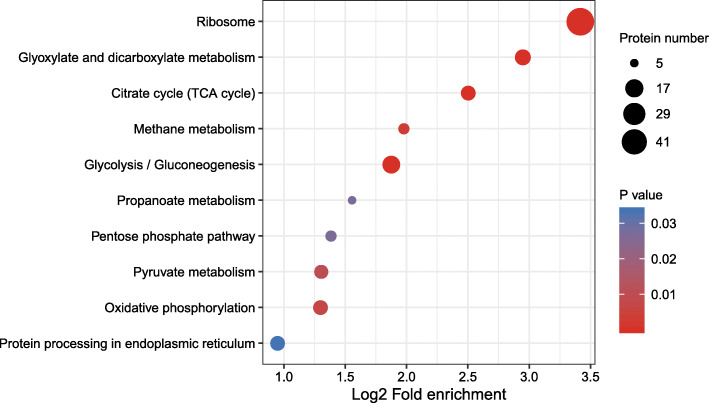
Enrichment bubble plot of *S*. *sanghuang* proteins corresponding to modification sites in the KEGG pathways

To further analyse the proteins and their functions, we performed functional enrichment analysis of the obtained malonylome by GO, KEGG pathway and protein domain analyses (Additional file 2: Table S5, Additional file 2: Table S6). Proteins associated with structural components of the ribosome were highly enriched by functional analysis of GO terms (Additional file 2: Table S4). Based on GO cellular component classification, proteins located in the ribosomal subunit, ribosome, large ribosomal subunit, small ribosomal subunit, and cytosol were more likely to be malonylated (Additional file 1: Fig. S2). Domain enrichment studies indicated that these proteins were the core histone H2A/H2B/H3/H4, proteasome, beta-ketoacyl synthase, 1-cys peroxiredoxin, acyl transferase domain, isocitrate/isopropyl malate dehydrogenase, and oxidoreductase flavin adenine dinucleotide (FAD)-binding domain proteins (Additional file 1: Fig. S3). These enriched domains play a crucial role in glycolysis, polysaccharide synthesis and the tricarboxylic acid (TCA) cycle in *S. sanghuang*. To probe the process of malonylation regulation, we further performed enrichment analysis of proteins corresponding to malonylation modification sites in KEGG pathways (Fig. 4). Several pathways of the enriched proteins in the ribosome, glucuronide and dicarboxylic acid metabolism, TCA cycle, glycolysis/gluconeogenesis and pyruvate metabolism pathways were enriched. In conclusion, malonylated proteins were enriched in several types of proteins and pathways, suggesting a pivotal role of lysine malonylation in the metabolism of *S. sanghuang.*

### PPI network of malonylated proteins in *S*. *sanghuang*

**Fig. 5:**
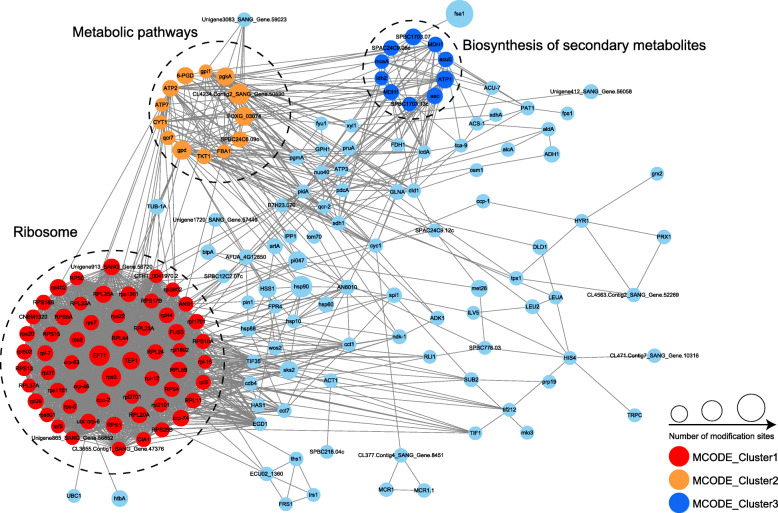
Interaction network of the identified proteins in *S*. *sanghuang*

To determine how the identified proteins were associated with multiple pathways, a PPI network was constructed. Ninety proteins were identified in the PPI database (Fig. 5, Additional file 2: Table S7), presenting a global view of how the identified malonyl proteins are involved in multiple pathways in *S. sanghuang*. Analysis of the STRING PPI network with Cytoscape identified three strongly correlated clusters of malonylated proteins, including those associated with ribosomes, metabolic pathways, and the biosynthesis of secondary metabolites in *S. sanghuang*. Overall, we conclude that malonylation is a critical PTM for proteins in *S. sanghuang* and helps in interactions and coordination with diverse pathways.

### Malonylated proteins associated with the biosynthesis of bioactive compounds in *S. sanghuang*

**Fig. 6:**
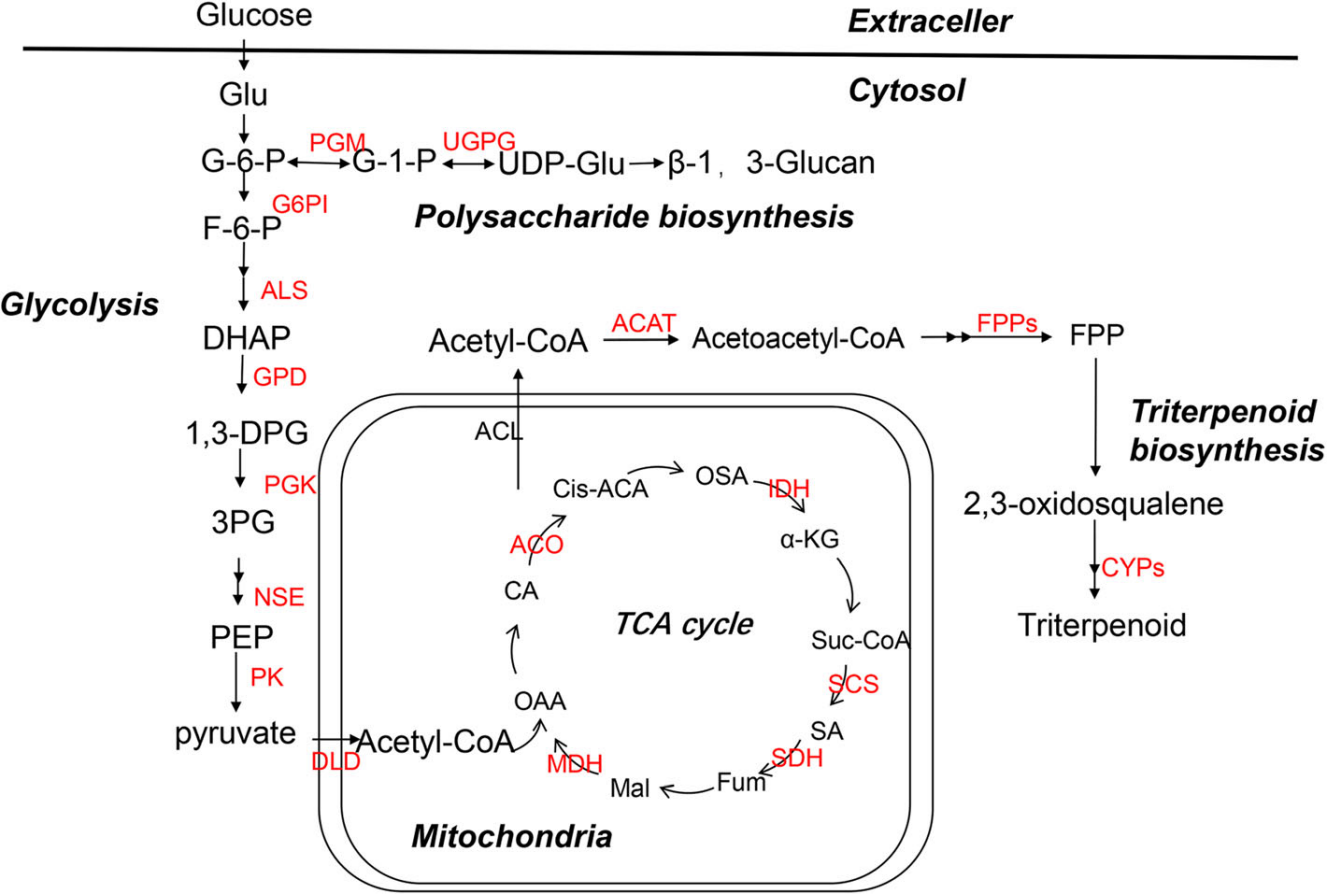
Biosynthesis of triterpenoid and polysaccharide in *S. sanghuang*. Malonylated proteins are highlighted in red. Additional file 2: Table S2 contains the enzyme annotation

**Fig. 7:**
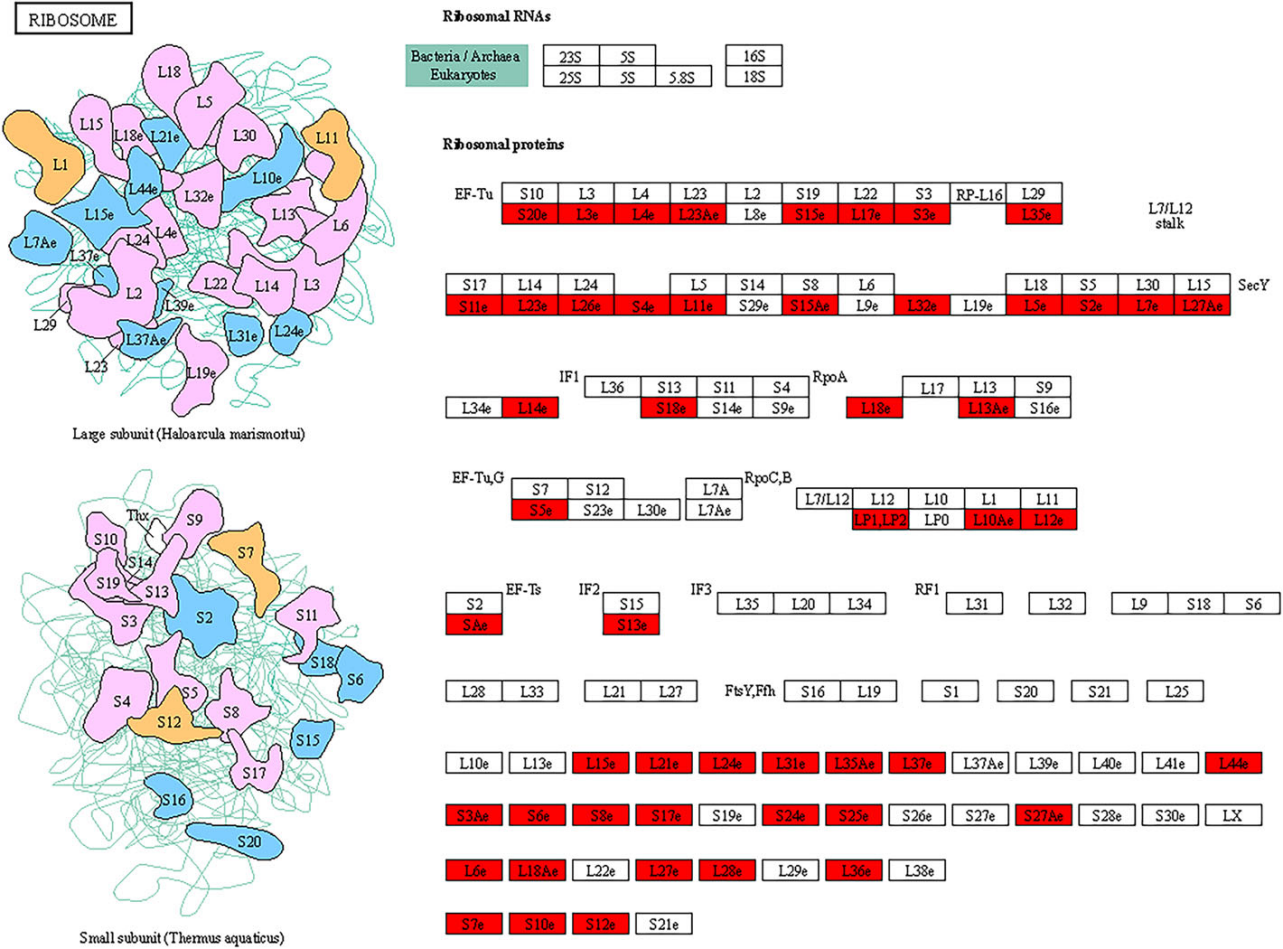
Malonylated modified sites on ribosomal proteins in *S. sanghuang*. The malonylated modified sites were highlighted in red

Malonylated proteins related to ribosomes, glucuronide and dicarboxylic acid metabolism, glycolysis/gluconeogenesis, the TCA cycle, methane metabolism, oxidative phosphorylation, and pyruvate metabolism were greatly enriched (Fig. 4). These findings suggested that the malonylation of lysine may be essential in the biosynthesis of bioactive compounds in *S. sanghuang.* To further confirm these findings, we analysed malonylated proteins associated with triterpene and polysaccharide biosynthesis in *S. sanghuang*. Consistent with these hypotheses, a total of 26 enzymes associated with triterpene and polysaccharide biosynthesis were found to be malonylated (Fig. 6, Additional file 2: Table S8). As shown in Fig. 6, a large number of enzymes were affected by malonylation in glycolysis and the TCA cycle, suggesting that malonylation may be associated with multiple levels of intracellular metabolism. Furthermore, our results also showed that 51 malonyl-modified proteins detected on ribosomes, such as ribosomal proteins L24, L13a, and S3, were closely linked to bioactive functions (Fig. 7).

## Discussion

As a widely used medicinal fungus, *S. sanghuang* has been known for many years worldwide. It has been reported that *S. sanghuang* is capable of producing many active substances, such as terpenes, flavonoids, and polysaccharides [5, 40–42]. However, the regulatory mechanism of the biosynthesis of these active compounds is still unclear. Lysine malonylation exists widely in eukaryotes and prokaryotes and has many metabolic regulatory functions. To investigate the role of lysine malonylation in the regulation of bioactive compound metabolism, we performed the first proteomic survey of lysine malonylation in *S. sanghuang*.

The metabolic processes of bioactive substances are related to secondary metabolism. Our malonyl analysis revealed a great number of malonylated proteins participating in secondary metabolism (Fig. 5), demonstrating the essential role of lysine malonylation in all these processes. Other types of PTMs such as acetylation and succinylation also participate in secondary metabolic processes in fungi [7, 43]. Similar to *S. sanghuang*, *Ganoderma lucidum* is also a widely used medicinal mushrooms worldwide [43]. It has been well documented that a large number of succinylated proteins are involved in the secondary metabolic process in *G. lucidum* [43]. Previous studies have shown that the secondary metabolism of *Fusarium graminearum* is regulated by acetylation [7]. These studies suggest that the secondary metabolic processes associated with the biosynthesis of bioactive substances are regulated by multiple protein modifications.

Polysaccharides are among the main bioactive substances produced by medicinal mushrooms [43]. In *G. lucidum*, 9 kinds of enzymes associated with polysaccharide biosynthesis have been found [44]. Among them, phosphoglucomutase (PGM) and UDP-glucose 6-dehydrogenase (UGDH) are succinyl-modified proteins in *G. lucidum* [43]. As shown in Fig. 6, PGM and UDP-glucosepyrophosphorylase (UGPG) were malonylated in *S. sanghuang*. To date, more than 700 kinds of medicinal mushrooms have been shown to produce bioactive polysaccharides [45]. These observations suggest that multiple PTMs, including malonylation and succinylation, participate in the regulation of polysaccharide biosynthesis in medicinal mushrooms.

Another major bioactive substance produced by *S. sanghuang* was triterpenoids. Previous studies have proven that triterpenoids are biosynthesized by the mevalonic acid (MVA) pathway [44]. As shown in Fig. 6, the first enzyme in the MVA pathway is acetyl-CoA acetyltransferase (ACAT), which converts acetyl-CoA to acetoacetyl-CoA. FPPs are crucial enzymes in the MVA pathway of triterpene metabolism [46]. Further modification of terpenes involves the introduction of acyl, aryl, or glycosyl groups, usually starting with oxidation catalysed by cytochrome P450 monooxygenases (P450s, also known as CYPs). P450s are ubiquitous in nature and are involved in fundamental biological pathways such as terpene biosynthesis [47–50]. All these key enzymes were detected by malonyl enrichment (Fig. 6). Thus, lysine malonylation plays a multilevel regulatory role in the biosynthesis of secondary metabolism enzymes.

Furthermore, different types of ribosomal proteins may have different biological activities. Ribosomal protein S5 (RPS5) is closely associated with liver fibrosis in Sprague-Dawley rats [51]. RPS13a plays a role in plant defence against *Verticillium dahliae* infection [52]. RPS3 protected cells in the substantia nigra against MPTP-induced oxidative stress in a mouse model of Parkinson’s disease [53] and RPL24 had time and dose-dependent effects on HepG-2 cell growth inhibition [54]. In addition, ribosomal synthesis and posttranslationally modified peptides (RiPPs) are an important family of bioactive products [55]. As shown in Fig. 7, a total of 51 ribosomal proteins were modified by malonylation. These findings all support the irreplaceable role of protein malonylation in the synthesis of bioactive substances.

## Conclusions

In this study, we found 714 lysine malonyl-modified residues in 255 proteins in *S. sanghuang*.

Malonylated proteins are involved in a variety of biological processes, especially in secondary metabolic pathways. Further analysis showed that a large number of enzymes involved in the biosynthesis of polysaccharides and triterpenoids were modified by malonylation. This research widens the scope of protein malonylation and provides a rich resource for exploring the physiological regulation of protein malonylation in *S. sanghuang*.

## Supplementary Information

**Additional file 1: Fig. S1.** The MS/MS spectra of examples of malonyl peptides. **Fig. S2.** GO-based enrichment analysis. **Fig. S3.** Domain enrichment analysis of the malonylproteins.

**Additional file 2: Table S1.** The identified malonylated sites in *S. sanghuang*. **Table S2.** Protein annotation analysis. **Table S3.** Analysis the sequence motifs of the malonylpeptides. **Table S4.** GO functional annotation of the malonylproteins. **Table S5.** The modified proteins based on KEGG pathway enrichment analysis. **Table S6.** The malonylated proteins based on domain enrichment analysis. **Table S7.** The proteins obtained from PPI network analysis. **Table S8.** The 26 enzymes associated with triterpene and polysaccharide biosynthesis were found to be malonylated. **Table S9.** BH correction (FDR) of the GO enrichment. **Table S10.** BH correction (FDR) of the KEGG enrichment. **Table S11.** BH correction (FDR) of the protein domain enrichment.

## Data Availability

All data generated or analysed during this study are included in this published article and its supplementary information files. The datasets supporting the results of this article are included within the article and additional files. The mass spectrometry proteomics data have been deposited to the ProteomeXchange Consortium (http://proteomecentral.proteomexchange.org) via the PRIDE partner repository with the dataset identifier PXD025835.
