# Supplementary figures and images for: Systematic analysis of the lysine malonylome in Sanghuangporus sanghuang
Source: BMC Genomics. 2021 Nov 19;22:840. doi: 10.1186/s12864-021-08120-0 (PMC8603570; doi:10.1186/s12864-021-08120-0)

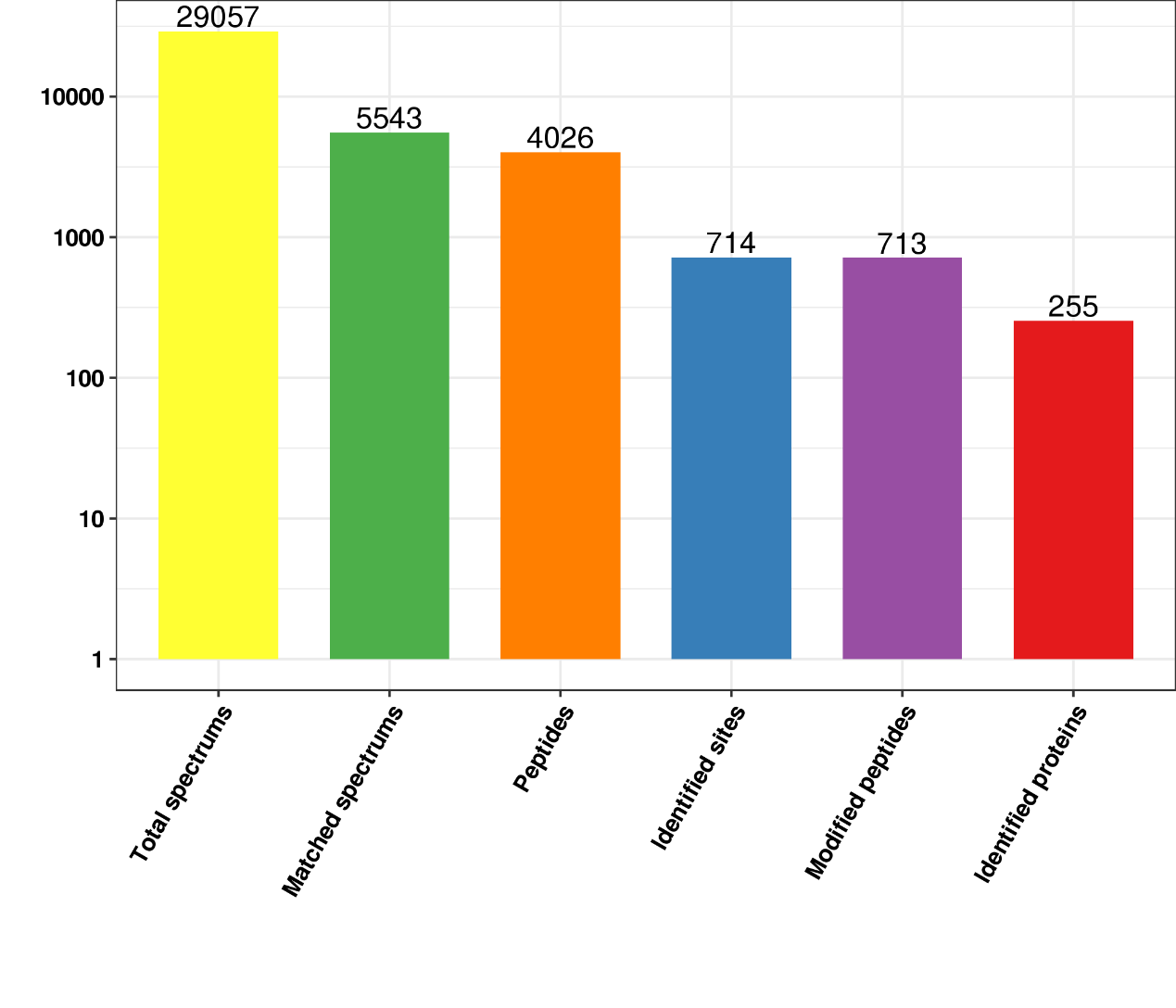
Figure S1. The MS/MS spectra of examples of malonyl peptides

Supplement: Supplementary file 1 — Additional file 1: Fig. S1. The MS/MS spectra of examples of malonyl peptides. Fig. S2. GO-based enrichment analysis. Fig. S3. Domain enrichment analysis of the malonylproteins. [file 12864_2021_8120_MOESM1_ESM.zip › Additional Files 1 Figure S1.docx]

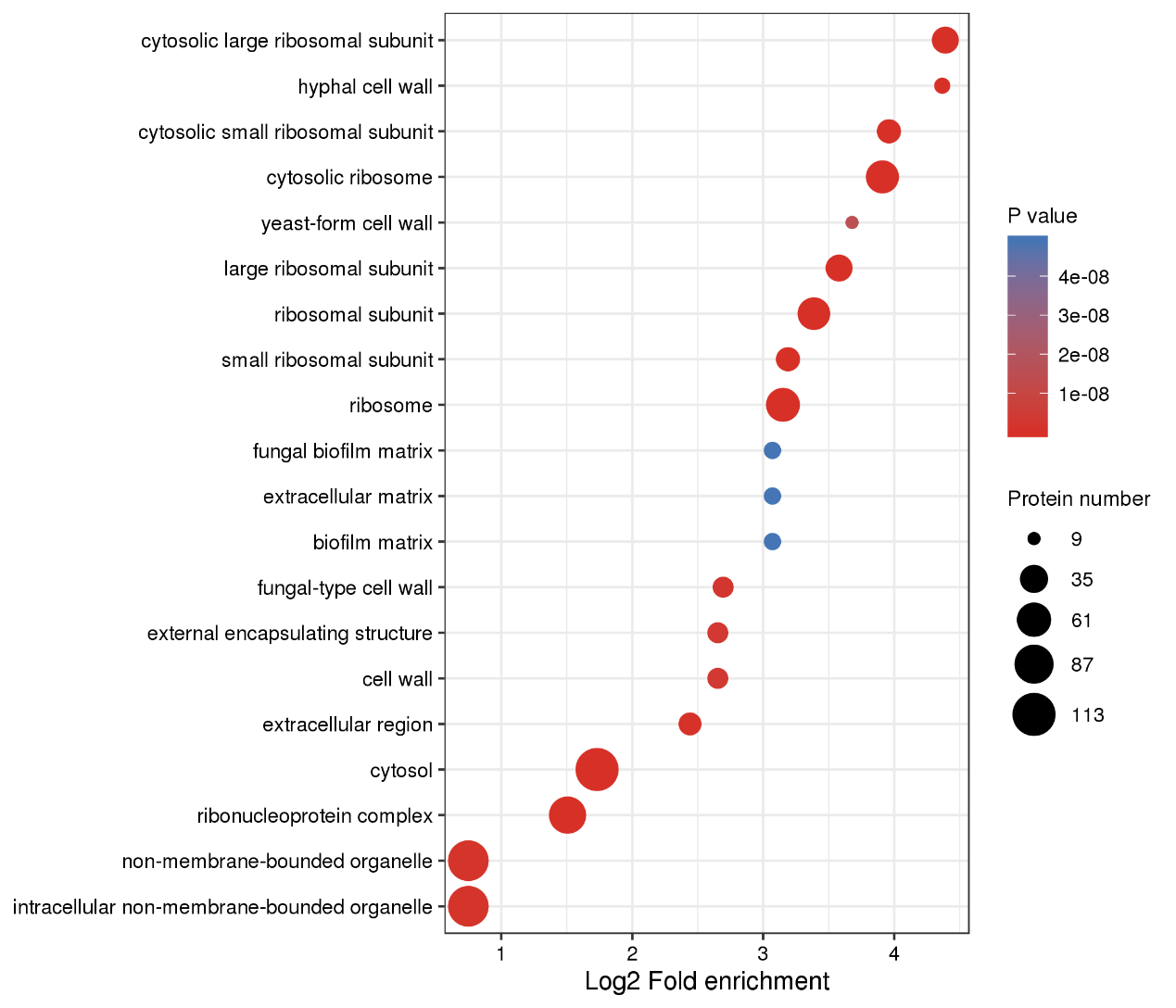


Figure S2. GO-based enrichment analysis in *S. sanghuang*

Supplement: Supplementary file 1 — Additional file 1: Fig. S1. The MS/MS spectra of examples of malonyl peptides. Fig. S2. GO-based enrichment analysis. Fig. S3. Domain enrichment analysis of the malonylproteins. [file 12864_2021_8120_MOESM1_ESM.zip › Additional Files 1 Figure S2.docx]

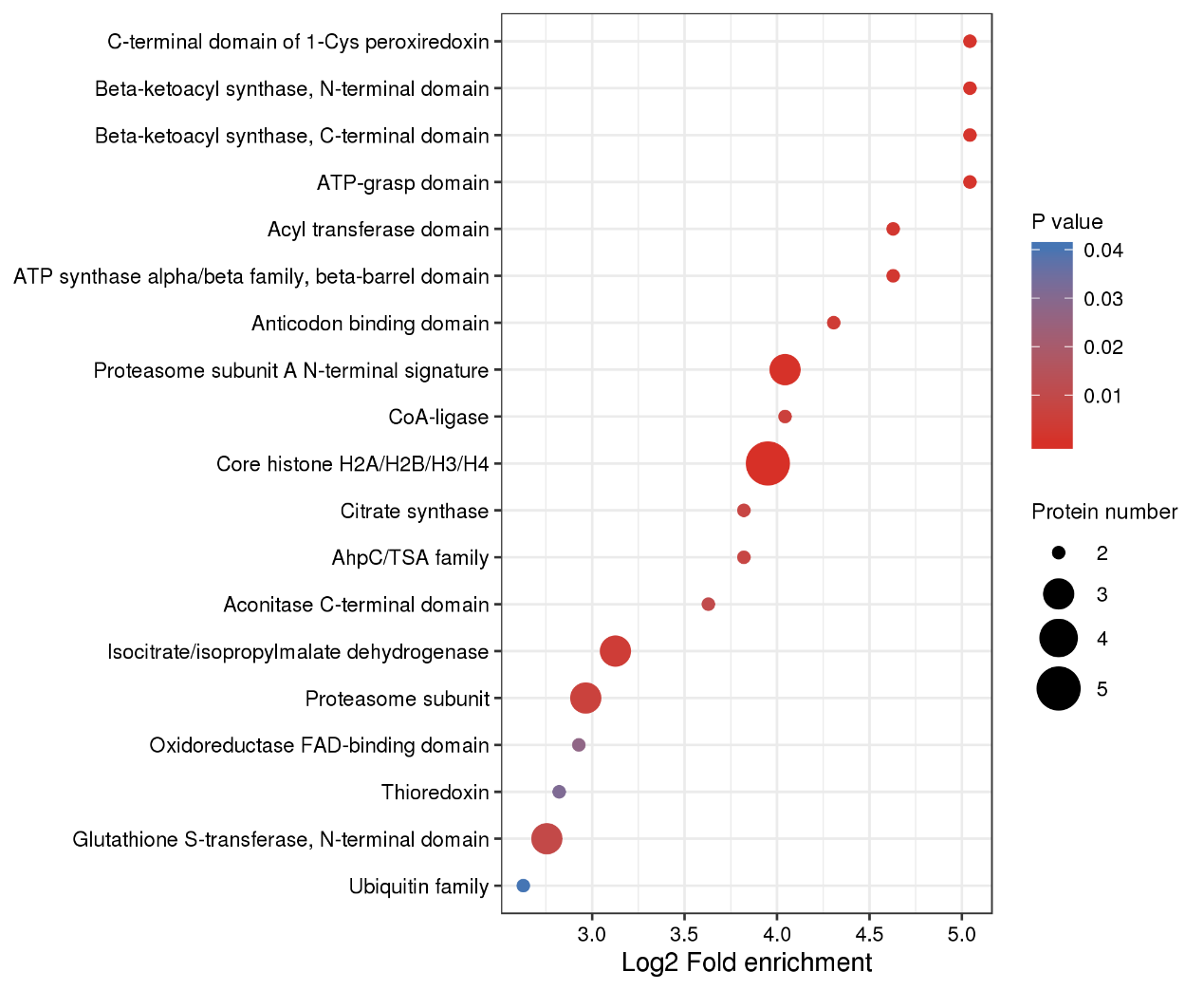


Figure S3. Domain enrichment analysis of the malonylproteins in *S. sanghuang*

Supplement: Supplementary file 1 — Additional file 1: Fig. S1. The MS/MS spectra of examples of malonyl peptides. Fig. S2. GO-based enrichment analysis. Fig. S3. Domain enrichment analysis of the malonylproteins. [file 12864_2021_8120_MOESM1_ESM.zip › Additional Files 1 Figure S3.docx]
